# Population-based prevalence of fetal alcohol spectrum disorder in Canada

**DOI:** 10.1186/s12889-019-7213-3

**Published:** 2019-06-28

**Authors:** Svetlana Popova, Shannon Lange, Vladimir Poznyak, Albert E. Chudley, Kevin D. Shield, James N. Reynolds, Margaret Murray, Jürgen Rehm

**Affiliations:** 1 Centre for Addiction and Mental Health, Institute for Mental Health Policy Research, 33 Russell Street, Toronto, ON M5S 2S1 Canada; 2 Dalla Lana School of Public Health, University of Toronto, 155 College Street, Toronto, ON M5T 3M7 Canada; 3 Factor-Inwentash Faculty of Social Work, University of Toronto, 246 Bloor Street W, Toronto, ON M5S 1V4 Canada; 4 Institute of Medical Science, University of Toronto, Faculty of Medicine, Medical Sciences Building, 1 King’s College Circle, Toronto, ON M5S 1A8 Canada; 5 Department of Mental Health and Substance Abuse, World Health Organization, 20 Avenue Appia, CH-1211 Geneva, Switzerland; 6 Department of Paediatrics and Child Health, University of Manitoba, 840 Sherbrook Street, Winnipeg, MB R3A 1S1 Canada; 7 Department of Biomedical and Molecular Sciences, School of Medicine, Queen’s University, 18 Stuart Street, Kingston, ON K7L 3N6 Canada; 8 National Institute on Alcohol Abuse and Alcoholism, National Institutes of Health, Bethesda, MD 20892 USA; 9 Institute of Clinical Psychology and Psychotherapy & Center of Clinical Epidemiology and Longitudinal Studies, Technische Universität Dresden, Chemnitzer Str. 46, 01187 Dresden, Germany; 10 Department of Psychiatry, University of Toronto, 250 College Street, Toronto, ON M5T 1R8 Canada

**Keywords:** Fetal alcohol spectrum disorder, Fetal alcohol syndrome, Prevalence, Prenatal alcohol exposure, Canada

## Abstract

**Background:**

Fetal alcohol spectrum disorder (FASD) is one of the most disabling potential outcomes of prenatal alcohol exposure. The population-based prevalence of FASD among the general population of Canada was unknown. The objective of this study was to determine the population-based prevalence of FASD among elementary school students, aged 7 to 9 years, in the Greater Toronto Area (GTA) in Ontario, Canada.

**Methods:**

This screening study used a cross-sectional, observational design utilizing active case ascertainment, along with retrospective collection of prenatal alcohol exposure information. Data collection involved two phases. Phase I consisted of taking growth measurements, a dysmorphology examination, and obtaining a history of behavioral and/or learning problems. Phase II consisted of a neurodevelopmental assessment, maternal interview, and behavioral observations/ratings by parents/guardians. Final diagnostic screening conclusions were made by consensus by a team of experienced multidisciplinary experts during case conferences, using the 2005 Canadian guidelines for FASD diagnosis. The prevalence of FASD was estimated, taking into consideration the selection rate, which was used to account for students who dropped out or were lost to follow-up during each phase. Monte Carlo simulations were employed to derive the confidence interval (CI) for the point estimates.

**Results:**

A total of 2555 students participated. A total of 21 cases of suspected FASD were identified. The prevalence of FASD was estimated to be 18.1 per 1000, or about 1.8%. Using a less conservative approach (sensitivity analysis), the prevalence of FASD was estimated to be 29.3 per 1000, or about 2.9%. Therefore, the population-based prevalence of FASD is likely to range between 2 and 3% among elementary school students in the GTA in Ontario, Canada.

**Conclusions:**

This study provides the first population-based estimate of the prevalence of FASD in Canada. The estimate is approximately double or possibly even triple previous crude estimates. FASD prevalence exceeds that of other common birth defects such as Down’s syndrome, spina bifida, trisomy 18, as well as autism spectrum disorder in Canada. More effective prevention strategies targeting alcohol use during pregnancy, surveillance of FASD, and timely interventions and support to individuals with FASD and their families are urgently needed.

**Electronic supplementary material:**

The online version of this article (10.1186/s12889-019-7213-3) contains supplementary material, which is available to authorized users.

## Background

Alcohol is a teratogen that readily crosses the placenta, interfering with the normal developmental progression of the embryo and fetus, and resulting in damage to the brain and other organs of the developing fetus. Fetal alcohol spectrum disorder (FASD) is one of the most disabling potential outcomes of prenatal alcohol exposure. A significant number of pregnancies are alcohol-exposed in Canada; it was recently estimated that approximately 10.0% of women in the Canadian general population consume alcohol while they are pregnant [1].

FASD includes three alcohol-related diagnoses: fetal alcohol syndrome (FAS), partial FAS (pFAS), and alcohol-related neurodevelopmental disorder (ARND) [2]. FASD is associated with a wide range of effects, including permanent brain damage, congenital anomalies, prenatal and/or postnatal growth restriction and characteristic sentinel facial features, along with cognitive, behavioral, emotional and adaptive functioning deficits [2, 3]. A recent systematic review identified over 400 disease conditions associated with FASD [3]. Some of these comorbid conditions (e.g., language, auditory, visual, developmental/cognitive, mental and behavioral problems) are highly prevalent among individuals with FAS, ranging from 50 to 91%, and significantly exceed the rates in the general population [3]. Furthermore, the neurodevelopmental impairments associated with FASD can, later in life, lead to academic failure, substance abuse, mental health problems, contact with law enforcement, and an inability to live independently and obtain and maintain employment [4]. Accordingly, FASD is recognized to impart a significant economic burden on society [5, 6].

Given that prenatal alcohol exposure has been recognized as the leading known preventable cause of birth defects and cause of developmental delay among Canadians [7], it is crucial to estimate the prevalence of FASD. Such estimates are vital for early detection, diagnosis, and intervention, as well as for informing policy-makers of the impact of FASD. However, a recent comprehensive literature review revealed that there have been no rigorous population-based epidemiological studies of FASD in Canada that used extensive outreach or other methods of active case ascertainment [8]. Therefore, to fill this gap, the objective of this study was to determine the population-based prevalence of FASD among elementary school students, 7 to 9 years of age, who attend public schools in the Greater Toronto Area (GTA) in Ontario, Canada.

## Methods

### Study design

This screening study, part of the World Health Organization International Collaborative Research Project on Child Development and Prenatal Risk Factors with a Focus on FASD, utilized a cross-sectional, observational design, using active case ascertainment (i.e., an epidemiological surveillance strategy in which cases are actively sought for examination and diagnosis), along with retrospective collection of prenatal alcohol exposure information. The study implemented a step-wise approach, where only those students meeting predetermined criteria proceeded to the subsequent phase. A full report of this study is available from Popova and colleagues [9].

### Sampling

This study sampled students, 7 to 9 years of age, who attended public schools in the GTA from September 2014 to June 2017. The GTA is comprised of five regional municipalities and is the most populous metropolitan area in Canada with a total population of 6.42 million in 2016, representing 18.3% of Canada’s population [10]. The GTA is representative of the general population of Ontario and Canada with respect to sex, age, and drinking patterns [11, 12]. In 2014–15, the GTA contained 1514 public elementary schools (1046 secular schools and 468 separate schools), administered by 10 district school boards, with a total enrolment of 642,014 [13]. It should be noted that the Canadian education system is characterized by inclusion, meaning that all children (including those with intellectual disabilities or developmental delays) attend a “mainstream” school, where they are given equal opportunity and support to study together with their typically developing peers in the same classroom.

The informed consent process was as follows: A letter from the principal of each school was sent home with the students, informing their parents/guardians of the study and its purpose. The written consent form was given to students to take home to their parents/guardians. One week later, a second round of consent forms was sent home with students who had not yet returned the completed form. Parents/guardians were given 2 weeks to return it. All students whose parents/guardians gave consent were informed about the study purpose and procedures and gave written assent to participate. All participating students received a small gift as a token of appreciation. The study protocol was approved by the Research Ethics Boards of the Centre for Addiction and Mental Health (165/2012) and of Health Canada / Public Health Agency of Canada (REB 2012–0052).

### Data collection

Data collection involved two phases: Phase I - pre-screening, and Phase II - screening (active case ascertainment). The purpose of the pre-screening phase was to identify students eligible for Phase II, which addressed three aspects of child development relevant to the diagnosis of FASD: 1) growth deficits; 2) sentinel facial features characteristic of FAS and pFAS; and 3) behavioral and/or learning problems. The screening phase (Phase II) included: 1) a neurodevelopmental assessment (please see Additional file 1 for a neurodevelopmental test battery used); 2) maternal interview; and 3) behavioral observations/ratings by parents/guardians via the Child Behavior Checklist (CBCL). The data collection was conducted by three independent research groups to minimize selection and researcher’s bias. The first group, comprised of trained research assistants, assessed growth and obtained histories of behavioral and/or learning problems. In addition, a trained dysmorphologist conducted dysmorphology assessment; the second group, comprised of qualified psychometrists and psychologists conducted the neurodevelopmental assessments. All assessments of students were conducted in schools during school hours. Biological mothers of students, who demonstrated deficits (defined as two standard deviations below the mean on a subtest) in a minimum of two domains assessed during the neurodevelopmental assessment, were invited for a telephone interview, conducted by trained interviewers. This threshold was set to increase the likelihood that all potential cases were identified, as impairment of a minimum of three domains is necessary for a FASD-specific diagnosis. The interview collected data from biological mothers on demographics and living environment, pregnancy history, alcohol use (during the past 30 days, lifetime drinking behavior, and drinking behavior prior to and following pregnancy recognition with the child in the study), nutrition during pregnancy, and tobacco and other drug use prior to and following pregnancy recognition. All study personnel were fully trained on the sensitive nature of alcohol use during pregnancy and its effects on the family. In addition, assessors were blinded as to which students were selected as controls and which students were selected because they met the eligibility criteria for Phase II.

### Typically developing control children

**Fig. 1:**
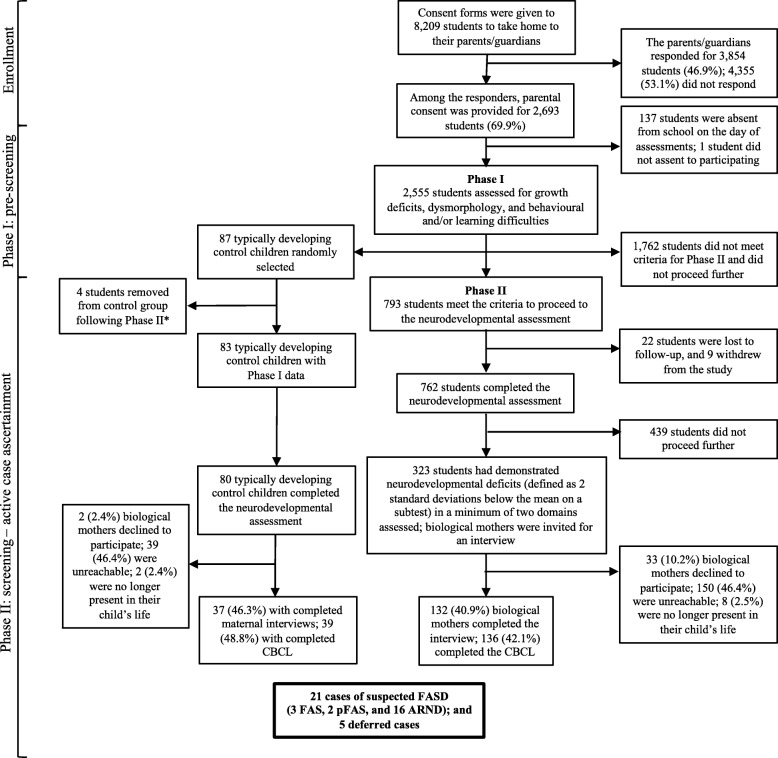
Sampling and recruitment methodology employed. ARND = alcohol-related neurodevelopmental disorder; CBCL = Child Behavior Checklist; FAS = fetal alcohol syndrome; FASD = fetal alcohol spectrum disorder; pFAS = partial fetal alcohol syndrome. *Four students were removed from the group of typically developing control children following Phase II because two students were found to have pre-existing neurodevelopmental disorders (1 had attention-deficit/hyperactivity disorder and 1 had speech delay), 1 was identified to have suspected ARND, and 1 was considered to be a deferred case

Typically developing control children (TDCC) were randomly selected from a list of all students who completed Phase I and who did not meet any of the criteria to qualify them to proceed to Phase II. These students underwent a complete assessment (i.e., physical, dysmorphological and neurodevelopmental assessments; maternal interviews to collect prenatal alcohol exposure history; and behavioural observations and ratings) to obtain normative data. For a detailed explanation of recruitment and sampling methodology, refer to Fig. 1 and the Additional file 1.

### Screening results: case conferences

The summary findings from the three independent research groups for all students who proceeded to Phase II and demonstrated deficits in a minimum of two neurodevelopmental domains, as well as for the TDCC, were discussed on a case-by-case basis during multidisciplinary case conferences attended by four experts in FASD diagnosis, the principal investigator, and the study coordinator (this group included psychologists, clinical geneticists, medical doctors, and epidemiologists). Final diagnostic conclusions were made by consensus, using the 2005 Canadian guidelines for FASD diagnosis [2].

The terms “deferred” and “suspected” were used as part of the screening. Deferred cases were those where prenatal alcohol exposure was identified, but where less than three central nervous system domains were considered impaired (thus, the diagnostic criteria for an FASD-specific diagnosis were not met at the time of the assessment). Suspected cases were those where prenatal alcohol exposure was identified and the diagnostic criteria for an FASD-specific diagnosis were met at the time of the assessment.

Please note that the development of this project was initiated in 2012 before the Canadian FASD diagnostic guidelines were updated in 2016 [14].

### Statistical analysis

Chi-square was used to test for categorical differences. Unpaired Student’s t-tests for normally distributed data or one-way analysis of variance (ANOVA) were used when comparing two or more groups, respectively, for differences in continuous measurements. If statistically significant differences were found by an ANOVA, a post-hoc analysis was performed using Tukey’s pairwise comparisons of means with equal variance. Significance was determined using an α of 0.05.

### Prevalence estimation

The prevalence of FASD, and each of the diagnostic categories (FAS, pFAS, ARND), was estimated taking into consideration the selection rate at each phase of data collection. It was assumed that there was no difference in the risk of FASD between those students whose parents/guardians provided consent to participate and those whose parents/guardians did not consent. As a sensitivity analysis, the possibility of cases of FAS and other FASD diagnoses among non-selected individuals (i.e., TDCC) was accounted for.

The 95% confidence intervals (CIs) were estimated using Monte Carlo-like simulations [15]. These intervals were based on set of 100,000 simulated FAS, pFAS, ARND, and FASD prevalence rates constructed using simulated estimates of (i) selection rates, (ii) the prevalence of FASD, pFAS, and ARND among children with a maternal interview, (iii) the prevalence of FAS among children with a neurodevelopmental assessment, and (iv) the prevalence of FAS, pFAS, ARND, and FASD among children who tested negative at each selection phase (for the sensitivity analysis only).

All statistical analyses were performed using Stata 15.1 [16]. See the Supplement for the formulas and additional details on the prevalence calculations.

Additional details in the methodology employed and formulas used for calculations can be found in the Additional file 1.

## Results

### Sampling and recruitment

Five of the 10 district school boards, representing four of the five regional municipalities, agreed to participate. Approval was sought from 71 school principals, of whom 40 allowed their school to participate. Participating schools belonged to both secular and separate school boards. From those schools that agreed to participate, consent forms were given to 8209 students to take home to their parents/guardians (as opposed to being mailed directly) – to ensure the privacy and confidentiality of those invited, schools were not permitted to provide parental contact information. A total of 3854 parents/guardians (46.9%) responded to the request for their child to participate in the study: 1161 (30.1%) refused to provide consent, and 2693 (69.9%) gave consent. On the days of Phase I assessments, 137 students were absent, resulting in 2556 students available for assessment. Of these, one student did not assent to participating. Therefore, in total, 2555 students were assessed for Phase I (growth, dysmorphology, and history of behavioral and/or learning problems). Facial photographs were taken of 1684 students (65.9%).

Of the 2555 students who participated in Phase I, 48.3% were male and had a mean age of 8.7 years (standard deviation [SD] = 0.9; age range: 6.4–10.8 years). The assessment revealed that 334 (42.1%) students had growth deficits (height and weight, and/or OFC at or below the 10th percentile) and/or at least two of the three characteristic sentinel facial features that discriminate between individuals with and without FAS/pFAS; 101 (12.7%) had growth deficits and/or at least two of the three characteristic sentinel facial features, along with behavioral and/or learning problems; and 358 (45.1%) had behavioral and/or learning problems, but no growth deficits or characteristic sentinel facial features. As such, 793 (31.0%) students were selected to proceed to Phase II.

Among 762 students who completed the neurodevelopmental assessment, 323 (42.4%) demonstrated neurodevelopmental deficits in a minimum of two domains. The biological mothers of these students were then invited for an interview. A total of 132 (40.9%) biological mothers completed the interview, and 136 (42.1%) parents/guardians completed the CBCL.

### Typically developing control children

In total, 87 children were randomly selected from the list of students who completed Phase I and who did not meet any of the criteria to proceed to Phase II. Eighty-four completed the neurodevelopmental assessment; maternal interviews were conducted for 41 (48.8%), and 43 (51.2%) parents/guardians completed the CBCL. Four students were removed from the group of TDCC following Phase II because two students were found to have pre-existing neurodevelopmental disorders (1 had attention-deficit/hyperactivity disorder and 1 had speech delay), 1 was identified to have suspected ARND, and 1 was considered to be a deferred case. A schematic diagram depicting the sampling and recruitment methodology employed is presented in Fig. 1.

### Suspected and deferred FASD cases

Final screening results revealed that 21 students met the criteria outlined in the 2005 Canadian guidelines for FASD diagnosis [2]: 3 students had suspected FAS, 2 had suspected pFAS and 16 had suspected ARND. In addition, 5 students were considered to be deferred cases (i.e., prenatal alcohol exposure was identified, but fewer than three central nervous system domains were found to be impaired at the time of assessment).

### Comparison of students with suspected FASD with typically developing control children

Students with suspected FASD did not differ from TDCC in terms of their sex, age, or ethnicity. Students with suspected FASD were more likely to be at or below the 10th percentile for height and OFC compared to TDCC *(p* < .001). As expected, significantly more students with suspected FASD had shorter PFLs (i.e., 2 SD below the mean) compared with TDCC (*p* < .001 for right PFL and *p* < .01 for left PFL). A smooth philtrum and narrow vermillion border of the upper lip (lip-philtrum guide scores of 4) were observed in 23.8 and 19.1% of students with suspected FASD, respectively (see Table 1 for the detailed results of Phase I assessments).

**Table 1:** Demographic characteristics and growth and dysmorphology measurements of screened students

	Students screened in Phase I (*n* = 2555)	Students eligible for Phase II (*n* = 817)	Students with deficits in 2+ neuro-developmental domains (*n* = 323^a^)	Students selected for case conference review (*n* = 66^b^)	Students with suspected FASD (*n* = 21)	Typically developing control children (*n* = 83)	Statistical test^c^	*p* value
Demographics								
Sex (% male)	48.3	55.2	58.8	50.0	52.4	59.0	*t* = 0.547	.586
Age (years) – mean (*SD*)	8.7 (0.9)	8.6 (0.9)	8.6 (1.0)	8.7 (1.0)	8.9 (0.8)	8.5 (0.8)	*t* = 1.859	.066
Range	6.4–10.8	6.7–10.6	6.9–10.4	6.9–10.3	7.6–10.4	6.5–10.5		
Ethnicity – *n* (%)							*X* = 7.279	.296
Caucasian	605 (23.7)	248 (30.4)	108 (33.4)	28 (42.4)	15 (71.4)	38 (45.8)		
African Canadian	244 (9.6)	73 (8.9)	41 (12.7)	9 (13.6)	1 (4.8)	3 (3.6)		
Eastern European	205 (8.0)	63 (7.7)	16 (5.0)	5 (7.6)	2 (9.5)	7 (8.4)		
Western European	394 (15.4)	124 (15.2)	50 (15.5)	11 (16.7)	3 (14.3)	16 (19.3)		
Chinese/Southeast Asian	313 (12.3)	79 (9.7)	30 (9.3)	3 (4.6)	0 (0.0)	3 (3.6)		
South Asian	353 (13.8)	95 (11.6)	31 (9.6)	4 (6.1)	0 (0.0)	8 (9.6)		
Other	437 (17.1)	135 (16.5)	47 (14.6)	6 (9.1)	0 (0.0)	8 (9.6)		
GROWTH MEASUREMENTS								
Height (cm) – mean (*SD*)	132.7 (7.9)	130.2 (8.2)	131.0 (8.7)	130.8 (9.1)	132.8 (7.7)	133.9 (7.2)	*t* = 0.633	.528
Height ≤ 10th percentile – *n* (%)	278 (10.9)	202 (24.7)	70 (21.7)	18 (27.3)	5 (23.8)	2 (2.4)	*X* = 12.226	< .001
Weight (kg) – mean (*SD*)	31.1 (8.0)	28.7 (7.8)	29.9 (8.6)	29.8 (9.2)	31.3 (9.5)	32.0 (8.5)	*t* = 0.346	.730
Weight ≤ 10th percentile – *n* (%)	272 (10.7)	202 (24.7)	65 (20.1)	17 (25.8)	4 (19.1)	7 (8.4)	*X* = 1.996	.158
OFC (cm) – mean (*SD*)	53.0 (1.7)	52.31 (1.9)	52.54 (1.9)	52.25 (1.9)	52.5 (2.2)	53.6 (1.4)	*t* = 2.942	.004
OFC ≤ 10th percentile – *n* (%)	256 (10.0)	254 (31.1)	79 (24.5)	20 (30.3)	5 (23.8)	0 (0.0)	*X* = 20.760	< .001
DYSMORPHOLOGY								
Right PFL (cm) – mean (*SD*)	2.51 (0.18)	2.48 (0.19)	2.50 (0.21)	2.44 (0.17)	2.40 (0.15)	2.51 (0.14)	*t* = 3.328	.001
Right PFL 2 *SD* below mean – *n* (%)	582 (22.8)	281 (34.4)	105 (32.6)	28 (42.4)	10 (47.6)	9 (10.8)	*X* = 15.180	< .001
Left PFL (cm) – mean (*SD*)	2.51 (0.17)	2.48 (0.18)	2.50 (0.20)	2.45 (0.17)	2.41 (0.16)	2.51 (0.13)	*t* = 2.658	.009
Left PFL 2 *SD* below mean – *n* (%)	562 (22.0)	268 (32.8)	96 (29.8)	28 (42.4)	9 (42.9)	11 (13.3)	*X* = 9.456	.002
Inner canthal distance (cm) – mean (*SD*)	2.88 (0.26)	2.82 (0.26)	2.82 (0.28)	2.74 (0.25)	2.84 (0.31)	2.83 (0.22)	*t* = 0.253	.801
Inner canthal distance ≤25th percentile – *n* (%)	912 (36.3)	358 (44.6)	148 (46.7)	39 (60.9)	10 (47.6)	38 (46.3)	*X* = 4.565	.335
Philtrum length (cm) – mean (*SD*)	1.18 (0.24)	1.19 (0.28)	1.20 (0.30)	1.24 (0.40)	1.17 (0.18)	1.26 (0.42)	*t* = 0.895	.373
Philtrum score on lip-philtrum guide – *n* (%)							*X* = 1.608	.658
1	175 (6.9)	47 (5.8)	15 (4.6)	2 (3.0)	0 (0.0)	1 (1.2)		
2	907 (35.5)	238 (29.1)	90 (27.9)	15 (22.7)	5 (23.8)	30 (36.1)		
3	1135 (44.4)	341 (41.7)	149 (46.1)	33 (50.0)	11 (52.4)	38 (45.8)		
4	327 (12.8)	182 (22.3)	65 (20.1)	16 (24.2)	5 (23.8)	14 (16.9)		
5	10 (0.4)	9 (1.1)	4 (1.2)	0 (0.0)	0 (0.0)	0 (0.0)		
Vermillion border score on lip-philtrum guide – *n* (%)							*X* = 1.620	.655
1	300 (12.0)	66 (8.2)	27 (8.5)	3 (4.7)	1 (4.8)	2 (2.4)		
2	1135 (45.2)	323 (40.3)	134 (42.4)	21 (32.8)	9 (42.9)	35 (42.7)		
3	926 (36.9)	315 (39.3)	124 (39.2)	31 (48.4)	7 (33.3)	36 (43.9)		
4	143 (5.7)	93 (11.6)	29 (9.2)	9 (14.1)	4 (19.1)	9 (11.0)		
5	5 (0.2)	5 (0.6)	2 (0.6)	0 (0.0)	0 (0.0)	0 (0.0)		

*FASD* fetal alcohol spectrum disorder, *PFL* palpebral fissure length, *SD* standard deviation

^a^Out of 786 students who completed the neurodevelopmental assessment in Phase II

^b^Selected out of 323 students who demonstrated deficits in a minimum of two neurodevelopmental domains, along with 84 typically developing control children (total 407 cases)

^c^Comparing students with suspected FASD with typically developing control children

**Fig. 2:**
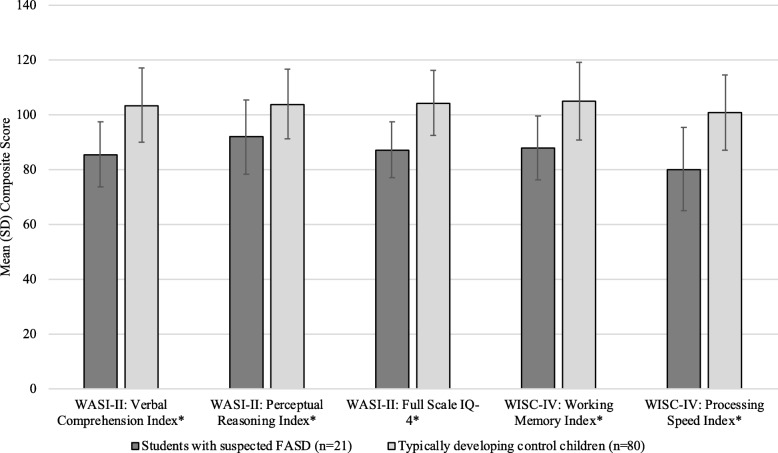
Mean (SD) composite scores on the WASI-II and WISC-IV among students with suspected FASD and TDCC. FASD = fetal alcohol spectrum disorder; TDCC = typically developing control children; WASI-II = Wechsler Abbreviated Scales of Intelligence, 2nd edition; WISC-IV = Wechsler Intelligence Scale for Children, 4th edition. *p < .001

**Fig. 3:**
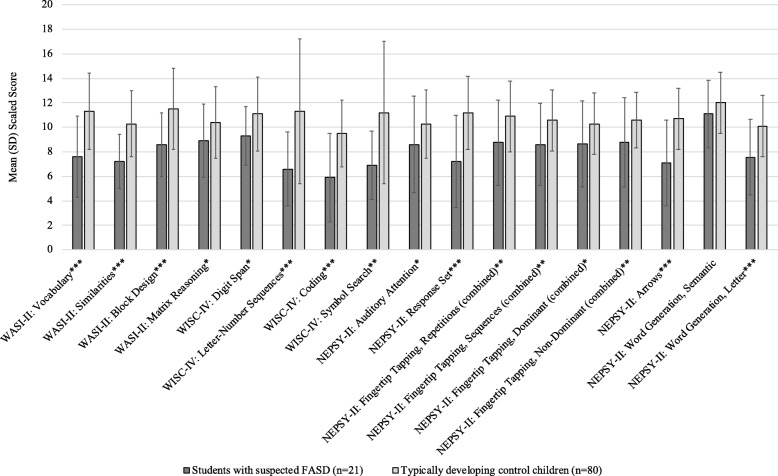
Mean (SD) scaled scores on the subtests of the WASI-II, WISC-IV and NEPSY-II among students with suspected FASD and TDCC. FASD = fetal alcohol spectrum disorder; TDCC = typically developing control children; WASI-II = Wechsler Abbreviated Scales of Intelligence, 2nd edition; WISC-IV = Wechsler Intelligence Scale for Children, 4th edition; NEPSY-II = A Developmental Neuropsychological Assessment, 2nd edition. *p < .05. **p < .01. ***p < .001

Neurodevelopmental assessment data revealed that, compared with typically developing control students, students with suspected FASD were characterized by lower scores on IQ (*p* < .001), verbal comprehension (*p* < .001), perceptual reasoning (*p* = .002), working memory (*p* < .001) and processing speed (*p* < .001), as per the composite scores of the WASI-II and WISC-IV (Fig. 2). Furthermore, the standard scores on all but one of the subtests (NEPSY-II: Word Generation, Semantic, which measures language) were statistically significantly lower among students with suspected FASD compared with TDCC (Fig. 3).

**Fig. 4:**
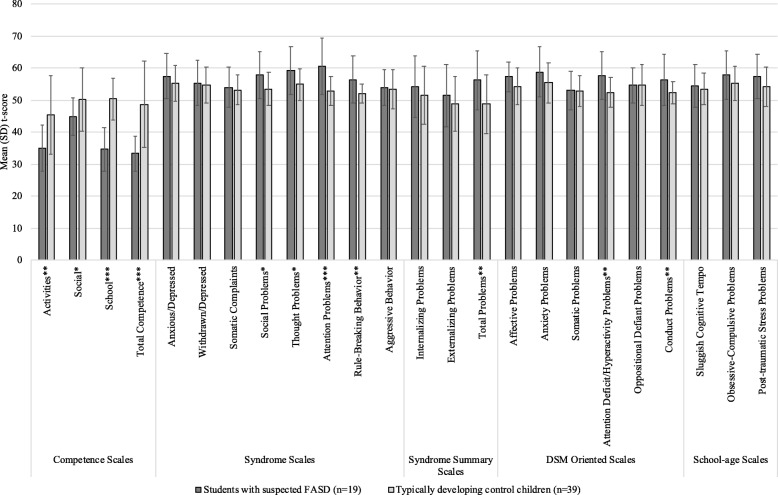
Mean (SD) t-score on the scales of the CBCL among students with suspected FASD and TDCC. CBCL = Child Behavior Checklist; FASD = fetal alcohol spectrum disorder; TDCC = typically developing control children. *p < .05. **p < .01. ***p < .001

As depicted in Fig. 4, students with suspected FASD scored significantly higher than TDCC on the Social Problems (*p* = .010), Thought Problems (*p* = .012), Attention Problems (*p* < .001) and Rule-Breaking Behavior (*p* = .002) Syndrome scales; Total Problems Syndrome summary scales (*p* = .006); and Attention Deficit/Hyperactivity Problems (*p* = .001) and Conduct Problems (*p* = .009) DSM-Oriented scales on the CBCL. Furthermore, TDCC scored significantly higher than students with suspected FASD on all Competence scales (Activities [*p* = .001], Social [*p* = .034], School [*p* < .001] and Total Competence [*p* < .001]).

It is important to note that students with suspected FASD were more likely than TDCC to have composite scores on the WASI-II and WISC-IV that were 1 to 2 SDs below the mean. Overall, considerably more students with suspected FASD had scores 1.5 SDs below the mean or lower on the Verbal Comprehension Index, Perceptual Reasoning Index, Full-Scale IQ-4, Working Memory Index and Processing Speed Index, compared with TDCC, while considerably more TDCC had scores 1.5 SDs above the mean or higher on these indices, compared with students with suspected FASD.

### Maternal characteristics

The mothers of students with suspected FASD did not differ significantly from mothers of TDCC with respect to their age, ethnicity, marital status, or employment status at the time of pregnancy with the child who participated in the study. However, mothers of students with suspected FASD had lower levels of education than mothers of TDCC at the time of pregnancy (*p* < .01). They were also more likely than mothers of TDCC to have smoked tobacco 68.4% vs. 18.9%, respectively, *p* < .001) and/or used marijuana or hashish (68.4% vs. 27.0%, respectively, *p* < .003) prior to pregnancy recognition. Among mothers of students with suspected FASD, only 63.2% of pregnancies were planned compared with 83.8% among mothers of TDCC (although this difference was not statistically significant). None of the mothers reported having a current drinking problem or ever having sought help for a drinking problem. All mothers of students with suspected FASD reported alcohol consumption prior to pregnancy recognition (high-risk levels: 63.2%, and some risk levels: 36.8%). Only 10.5% mothers of students with suspected FASD reported alcohol consumption following pregnancy recognition (some-risk levels only).

### Estimated prevalence of FASD

As per the main analysis, the prevalence of suspected FAS was estimated to be 1.2 per 1000 (95% CI: 0.0–2.8 per 1000), suspected pFAS was estimated to be 2.0 per 1000 (95% CI: 0.0–5.1 per 1000), and suspected ARND was estimated to be 15.0 per 1000 (95% CI: 8.1–22.7 per 1000). The overall FASD prevalence was estimated to be 18.1 per 1000 (95% CI: 10.8–26.3 per 1000) or 1.8% (Table 2).

**Table 2:** Prevalence of FASD (per 1000) among elementary school students in the Greater Toronto Area, Canada

FASD diagnostic categories	Total number of suspected cases	Main analysis			Sensitivity analysis		
		Prevalence	95% CI		Prevalence	95% CI	
			LE	UE		LE	UE
Suspected FAS	3	1.2	0.0	2.8	1.2	0.0	2.8
Suspected pFAS	2	2.0	0.0	5.1	2.0	0.0	5.1
Suspected ARND	16	15.0	8.1	22.7	26.1	9.6	52.8
Suspected FASD	21	18.1	10.8	26.3	29.3	12.4	56.2

*ARND* alcohol-related neurodevelopmental disorder, *CI* confidence interval, *FAS* fetal alcohol syndrome, *FASD* fetal alcohol spectrum disorder, *LE* lower estimate, *PE* prevalence estimate, *pFAS* partial fetal alcohol syndrome, *UE* upper estimate

### Sensitivity analysis

A sensitivity analysis was performed to account for the possibility of cases of FASD among non-selected individuals (i.e., among TDCC). As such, this scenario included one case of suspected ARND found among the TDCC. Based on this scenario, the prevalence of suspected FAS was estimated to be 1.2 per 1000 (95% CI: 0.0–2.8 per 1000), suspected pFAS 2.0 per 1000 (95% CI: 0.0–5.1 per 1000), and suspected ARND 26.1 per 1000 (95% CI: 9.6–52.8 per 1000). The overall FASD prevalence was estimated to be 29.3 per 1000 (95% CI: 12.4–56.2 per 1000) or 2.9% (Table 2).

## Discussion

This study provides the first population-based estimate of the prevalence of FASD among elementary school students (7 to 9 years of age) in Canada, which is generalizable to the general population of large urban areas of Canada. The prevalence ranges between approximately 2 and 3%, which is roughly double or possibly even triple previous crude estimates of 10 per 1000 or 1% (adopted for Canada from the United States) [17] and 7.9 per 1000 or about 0.8%, based on statistical modelling using country-specific indicators [8]. The estimated FASD prevalence exceeds that of other common birth defects such as Down’s syndrome, anencephaly, spina bifida, trisomy 18, as well as autism spectrum disorder in Canada.

This study used the most reliable approach to estimating FASD prevalence—active case ascertainment. It has primary advantages over other approaches, namely, representativeness of data by studying an entire community/population, a high chance of accurate diagnosis of FASD by clinical specialists, and elimination of self-selection biases [18]. Given these advantages, active case ascertainment is known to produce the most accurate FASD prevalence estimates [18].

These findings are in line with recent estimates in the United States, where the prevalence of FASD among the general population was estimated to be between 1 and 5%, using a conservative approach to estimation [19]. However, the current Canadian estimates of the prevalence of FASD are lower than recent estimates reported for some other countries, most likely due to the lower rates of alcohol consumption overall in Canada, as well as among pregnant women. For example, the prevalence of FASD in Croatia was estimated to be 4–7% [20, 21]; in Italy 4–5% [22, 23]; and in South Africa 6–21% [24, 25].

The results of this study have clear implications for both clinicians and researchers — namely that many cases of FASD are either missed or misdiagnosed (none of the identified children in this study were previously diagnosed with FASD); timely interventions and supports should be made available for children with FASD and their families; improved prevention efforts targeting prenatal alcohol use are needed; and it is necessary to establish universal surveillance systems for FASD and prenatal exposure to alcohol. Moreover, given that the current study estimated the prevalence of FASD among a diverse sample of elementary school students in the GTA, the findings emphasize that FASD is not restricted to disadvantaged groups, but, rather, that it occurs throughout society, regardless of socio-economic status, education, or ethnicity.

However, it should be acknowledged that the prevalence found in this study is likely underestimated for a number of reasons. First, the participation rate was lower than desired. Although two rounds of consent forms were distributed, it was not possible to ensure that all parents/guardians received the forms because, as specified above, they were given to students to take home rather than being mailed directly. As a result, it is not known whether the parents/guardians, who did not respond, actually received the form from their children or if these parents were ‘soft’ refusals, where they received the form but did not wish to participate in the study. Second, there was a potential for self-selection bias (i.e., the parents’ decision to allow their child to participate in the study may have reflected some inherent bias in the characteristics of their child). Third, in some cases, the teachers were not available to provide referrals, and it is also possible that some parents/guardians were not willing to identify behavioral and/or learning difficulties in their children due to social desirability bias. Fourth, information on prenatal exposure to alcohol was obtained via self-reports of the biological mothers, which was likely underreported due to social desirability and recall bias [26]. Fifth, only 40.9% of biological mothers agreed to be interviewed and alternative sources of information regarding maternal alcohol use were not sought as per a stipulation of the Research Ethics Boards. Therefore, some cases of pFAS and ARND potentially could be missed. Lastly, it is important to emphasize that the five deferred cases identified in this study are still at-risk for an FASD diagnosis given that additional deficits may emerge later in life.

With respect to the neurodevelopmental assessments, the findings are consistent with other research, demonstrating that children with FASD have lower composite scores for IQ, verbal comprehension, perceptual reasoning, working memory, processing speed and elevated externalizing behavioural problems, compared with typically developing control children [27, 28]. However, the finding that the mean scores of students with suspected FASD, as a group, did not demonstrate profound deficits should be interpreted with caution. Although 2 SDs below the mean is used as the cut-point in the 2005 Canadian guidelines [2], when looking at the individual performances of students with suspected FASD, many of them had scores 1.5 SDs below the mean or lower on the WASI-II and WISC-IV composite scores. Such scores are considered to be clinically relevant, as they place the individual in the borderline range of intellect/cognitive ability and are highly significant with respect to daily functioning. Thus, such individuals may require remedial educational support and interventions.

## Conclusions

The results of the current study clearly show that FASD must be considered as a serious preventable public health problem in Canada and support the need to improve prevention initiatives around alcohol use among not only pregnant women, but among all women of childbearing age, as well as the need to provide support to affected individuals and their families. As such, efforts are needed to broadly build awareness about the harmful effects of alcohol use during pregnancy; promote the routine discussion of alcohol use and the related risks with pregnant women and women of childbearing age; identify and provide support to pregnant women with alcohol use problems; and plan and deliver postpartum support for new mothers [29]. Such strategies are in agreement with the World Health Organization guidelines for the identification and management of substance use and substance use disorders in pregnancy [30].

## Additional file

Additional file 1: WHO_FASD_PrevalenceCanada_BMCPH Appendix_Aug29_18(.doc). Appendix. Details of data collection, participant recruitment and calculations for population-based prevalence estimates. (DOCX 43 kb)

## Data Availability

The datasets generated and/or analyzed during the current study are not publicly available due to the highly sensitive nature of the study and to protect the identity of the diagnosed students and their mothers.

## Abbreviations

ACA: active case ascertainment
ANOVA: one-way analysis of variance
ARBD: alcohol-related birth defects
ARND: alcohol-related neurodevelopmental disorder
CBCL: Child Behavior Checklist
CI: confidence interval
FAS: Fetal Alcohol Syndrome
FASD: fetal alcohol spectrum disorder
GTA: Greater Toronto Area
pFAS: partial Fetal Alcohol Syndrome
SD: standard deviation
TDCC: typically developing control children
