# Supplementary material for: Population-based prevalence of fetal alcohol spectrum disorder in Canada
Source: BMC Public Health. 2019 Jun 28;19:845. doi: 10.1186/s12889-019-7213-3 (PMC6599312; doi:10.1186/s12889-019-7213-3)
Supplement: Supplementary file 1 — WHO_FASD_PrevalenceCanada_BMCPH Appendix_Aug29_18(.doc). Appendix. Details of data collection, participant recruitment and calculations for population-based prevalence estimates. (DOCX 43 kb) [file 12889_2019_7213_MOESM1_ESM.docx]

Appendix

Population-based Prevalence of Fetal Alcohol Spectrum Disorder in Canada

Svetlana Popova, Shannon Lange, Vladimir Poznyak, Albert E. Chudley, Kevin D. Shield, James N. Reynolds, Margaret Murray, & Jürgen Rehm

**Methods**

The methodology for determining the prevalence of FASD was developed in consultation with leading international researchers (see “Acknowledgments” section) under the guidance of the World Health Organization, as well as the National Institute on Alcohol Abuse and Alcoholism (USA), and was based on advances in the diagnosis of FASD in Canada.^1^

Recruitment

Permission to conduct external research was solicited from all 10 school boards. Within the boards that agreed to participate, schools were randomly selected, with preference given to schools with the largest enrolment. Consent forms were given to all students in grades 2, 3, and 4 to take home to their parents/guardians. All students whose parents/guardians gave consent were also assented.

Data collection

Phase I: pre-screening.

***Physical development.*** Height, weight and occipitofrontal circumference (OFC) were measured, and percentiles were calculated using the respective Centers for Disease Control and Prevention clinical growth charts (<https://www.cdc.gov/growthcharts/clinical_charts.htm>).

***Dysmorphology assessment.*** The primary method of conducting the dysmorphology assessment was by direct examination of facial (short palpebral fissures, smooth or flattened philtrum, and thin vermilion border of the upper lip) and other characteristic features (e.g., ptosis, epicanthal folds, strabismus, and “hockey stick” palmar crease). Available normative data were used to compare the palpebral fissure length (PFL)^2^ and inner canthal distance^3^ measurements to calculate percentile rank. As a secondary method (i.e., to validate the direct examination), facial photographs were taken and analyzed using the FAS Facial Photographic Analysis Software, version 2.0.^4^

***Behavioral and/or learning problems.*** Students with behavioral and/or learning difficulties (e.g., maladaptive behavior, inattention, hyperactivity, and learning problems) were identified by teachers and/or parents/guardians.

**Proceeding to Phase II screening.**

Students were selected to proceed to Phase II if they met one or more of the following criteria:

1. height and weight at or below the 10th percentile;
2. OFC at or below the 10th percentile;
3. presence of at least two of the three characteristic sentinel facial features that discriminate between individuals with and without FAS:
4. short palpebral fissures (2 standard deviations below the mean; at or below the 3rd percentile),
5. smooth or flattened philtrum (4 or 5 on the 5-point Likert scale of the lip-philtrum guide), and
6. thin vermilion border of the upper lip (4 or 5 on the 5-point Likert scale of the lip-philtrum guide);^5^ and
7. existing behavioral and/or learning problems, neurodevelopmental disorder(s) (e.g., attention-deficit hyperactivity disorder, conduct disorder, autism spectrum disorder), and/or learning disabilities.

**Typically developing control children.** Typically developing control children were randomly selected from a list of all students who completed Phase I and who did not meet any of the criteria to qualify them to proceed to Phase II (see above). These students underwent a complete assessment in Phase II. Students whose mothers reported alcohol consumption during pregnancy at a level considered to be “high risk” (see definition in Supplement) or who were reported to have a pre-existing neurodevelopmental disorder were excluded from the control group.

Phase II: screening – active case ascertainment.

***Neurodevelopmental assessment.*** A team of qualified psychometrists conducted the neurodevelopmental assessment, which included tests of attention, executive function, general cognition, language, processing speed, sensorimotor working memory, and visuospatial processing. The following neurodevelopmental test battery was used: the Wechsler Abbreviated Scales of Intelligence, second edition (WASI-II; subtests: block design, matrix reasoning, similarities, and vocabulary);^6^ four subtests from the Wechsler Intelligence Scale for Children, fourth edition (WISC-IV; coding, digit span [forward and backward], letter-number sequencing, and symbol search);^7^ and four subtests from the Developmental Neuropsychological Assessment, second edition (NEPSY-II; arrows, auditory attention and response set, fingertip tapping, and word generation).^8^

***Maternal interview and behavioral observations/ratings.*** A 30-minute semi-structured telephone interview with the biological mother was requested for students who demonstrated deficits (defined as two standard deviations below the mean on a subtest) in a minimum of two domains assessed during the neurodevelopmental assessment. This threshold was set to increase the likelihood that all potential cases were identified, as impairment of a minimum of three domains is necessary for a FASD-specific diagnosis. The interview collected data on demographics and living environment, pregnancy history, alcohol use (during the past 30 days, lifetime drinking behavior, and drinking behavior prior to and following pregnancy recognition with the child in the study), nutrition during pregnancy, and tobacco and other drug use prior to and following pregnancy recognition. In addition, the mother was asked to complete the Child Behavior Checklist (CBCL), a well-established standardized parent/caregiver questionnaire used to evaluate social competencies and behavioral problems in children. In cases where the biological mother was no longer present in the child’s life, the student’s biological father or legal guardian was asked to complete the CBCL.

Definitions

The terms “deferred” and “suspected” were used as part of the screening. Deferred cases were those where prenatal alcohol exposure was identified, but where less than three central nervous system domains were considered impaired (thus, the diagnostic criteria for an fetal alcohol spectrum disorder [FASD]-specific diagnosis were not met at the time of the assessment). Suspected cases were those where prenatal alcohol exposure was identified and the diagnostic criteria for an FASD-specific diagnosis were met at the time of the assessment. As per the opinion of the multidisciplinary team of experts in FASD diagnosis and in alignment with the revised Canadian FASD diagnostic guidelines,^9^ prenatal alcohol exposure was considered to pose “high risk” if the biological mother reported two or more binge drinking episodes (four or more standard drinks on a single occasion) or seven or more standard drinks within one week. Prenatal alcohol exposure was considered to pose “some risk” if the biological mother reported alcohol consumption, but at a level considered to be lower than high-risk.

Prevalence estimation

For the main scenario, it was assumed that there was no difference in the risk of FASD between those students whose parents/guardians provided consent to participate and those whose parents/guardians did not consent. As described in the Methods section of this paper, the study used a step-wise approach to estimate the prevalence of FASD and each of the diagnostic categories within the spectrum (FAS, pFAS and ARND), whereby only those students who met predetermined criteria proceeded to the next phase. Specifically, the selection rate (SR) for each diagnostic category was estimated based on the number of students in the sample (nt) and the number of students who met one or more of the criteria (indicators of FASD) (ni), as this formula shows:

[Formula 1]

$$SR=\frac{n_{i}}{n_{t}}$$

To estimate each prevalence, SRs were used to account for students who dropped out or were lost to follow-up during each phase. As such, the prevalence was estimated, taking into account the SRs of each phase (see Formulas 2 and 3). In the case of FAS, the prevalence (PFAS_G) did not account for the SR with respect to the maternal interviews (because FAS can be diagnosed without confirmation of prenatal alcohol exposure), whereas for other-FASD diagnoses, the prevalence (Pother-FASD_G) of suspected cases identified among those students for whom maternal interview data were available was estimated. Each prevalence figure was then multiplied by 1,000 (k) to transform the prevalence into population rates. See Formulas 2 and 3, where SRpI is the selection rate following Phase I; SRpII is the selection rate following Phase II; PFAS is the number of suspected cases of FAS; and Pother-FASD is the number of suspected cases of other-FASD (i.e., pFAS/ARND). The prevalence of FASD was then estimated by adding together PFAS_G and Pother-FASD_G.

[Formula 2]

$$P_{FAS\_G}= \mathrm{SR}_{\mathrm{pI}}\cdot P_{\mathrm{FAS}}\cdot k$$

[Formula 3]

$$P_{other-FASD\_G}= \mathrm{SR}_{\mathrm{pI}}\cdot\mathrm{SR}_{\mathrm{pII}} \cdot P_{other-FASD}\cdot k$$

During phase I, 2,555 students were assessed for growth deficits, dysmorphology, and behavioral and/or learning difficulties; of those assessed, 793 students met the criteria to proceed to neurodevelopmental assessment resulting in a SRpI of 31.0%. Subsequently, of the 762 students that completed the neurodevelopmental assessment (22 students were lost to follow-up, and 9 withdrew from the study after Phase I screening), 323 students demonstrated neurodevelopmental deficits in a minimum of two domains resulting in a SRpII of 42.4%.

Sensitivity analysis. As a sensitivity analysis, the possibility of cases of FAS and other-FASD diagnoses among non-selected individuals (i.e., typically developing control children) was accounted for. As such, the one case of suspected ARND found among the typically developing control children was included here. In total, 87 of the 1,762 students who did not meet the criteria to proceed to Phase II were randomly selected to proceed through all study phases. In this analysis, the prevalence of FAS (PFAS_C) and other-FASD diagnoses (Pother-FASD_C) among the sample of 41 typically developing control children (i.e., those for whom maternal interview data were available) was incorporated into the above-noted prevalence estimates of FAS and other FASD diagnoses (including FAS) among the general population, using Formulas 4 and 5.

[Formula 4]

$$P_{FAS\_SENS}= P_{FAS\_G}+P_{FAS\_C}\cdot(1-{(SR}_{\mathrm{pI}}))\cdot k$$

[Formula 5]

$$P_{other-FASD\_SENS}= P_{other-FASD\_G}+P_{other-FASD\_C}\cdot(1-{(SR}_{\mathrm{pI}}\cdot\mathrm{SR}_{\mathrm{pII}}))\cdot k$$

It should be noted that the four students who were removed from the group of typically developing control children following Phase II (because two students were found to have pre-existing neurodevelopmental disorders [1 had attention-deficit/hyperactivity disorder and 1 had speech delay], 1 was identified to have suspected ARND, and 1 was considered to be a deferred case) were included in the prevalence calculations.

Estimation of confidence intervals. The corresponding 95% confidence interval for each respective prevalence estimate was determined using a Monte Carlo methodology,^10^ by using the 2.5th and 97.5th percentiles of a distribution of prevalences comprised of 100,000 estimates generated by taking sets of samples for the uncertainty distributions of each of the lowest-level parameters.

**References**

1. Chudley AE, Conry J, Cook JL, Loock C, Rosales T, LeBlanc N. Fetal alcohol spectrum disorder: Canadian guidelines for diagnosis. CMAJ. 2005;172(5 suppl):S1-S21.
2. Clarren SK, Chudley AE, Wong L, Friesen J, Brant R. Normal distribution of palpebral fissure lengths in Canadian school age children. Can J Clin Pharmacol. 2010;17(1):e67-78.
3. Hall JG, Froster-Iskenius UG, Allanson JE. Handbook of normal physical measurements. New York, NY: Oxford University Press; 1989.
4. FAS facial photographic analysis software. Version 2.0 [computer program]. Seattle, WA: FAS Diagnostic & Prevention Network, University of Washington; 2012.
5. Astley SJ, Clarren SK. Measuring the facial phenotype of individuals with prenatal alcohol exposure: correlations with brain dysfunction. Alcohol Alcohol. 2001;36(2):147-159.
6. Wechsler D. Wechsler Abbreviated Scale of Intelligence–Second Edition (WASI-II). San Antonio, TX: PsychCorp;2011.
7. Wechsler D. Wechsler Intelligence Scale for Children–Fourth Edition (WISC-IV). San Antonio, TX: PsychCorp;2008.
8. Korkman M, Kirk U, Kemp S. NEPSY-II: A developmental neuropsychological assessment. San Antonio, TX: PsychCorp;2007.
9. Cook JL, Green CR, Lilley CM, et al. Fetal alcohol spectrum disorder: a guideline for diagnosis across the lifespan. CMAJ. 2016;188(3):191-197.
10. Graham C, Talay D. Stochastic Simulation and Monte Carlo Methods: Mathematical Foundations of Stochastic Simulation. Berlin, Germany: Springer; 2013.
